# Association of insulin, C-peptide and blood lipid patterns in patients with impaired glucose regulation

**DOI:** 10.1186/s12902-019-0400-5

**Published:** 2019-07-15

**Authors:** Shujin Wang, Guohong Li, Hong Zuo, Hua Yang, Lei Ma, Jia Feng, Yu Niu, Liming Ma, Songfang Liu, Ting Qi, Xufeng Liu

**Affiliations:** 1 Department of Endocrinology, Ninth Hospital of Xi’an, No. 151 South Second Ring Road, Xi’an, Shaanxi 710054 China; 2 Department of Clinical laboratory, Ninth Hospital of Xi’an, No. 151 South Second Ring Road, Xi’an, Shaanxi 710054 China

**Keywords:** Type 2 diabetes (T2DM), Impaired glucose regulation, Lipid metabolism, OGTT, HDL-C, TG, TC, LDL-C

## Abstract

**Background:**

To investigate any associations between blood glucose (BG) and lipid levels in patients with different glucose tolerance statuses, including type 2 diabetes (T2DM) and impaired glucose regulation (IGR) cases as well as normal glucose tolerance (NGT) individuals.

**Methods:**

A total of 354 participants were recruited to this study including 174 in the T2DM group, 112 in the IGR group and 68 in the NGT group. We compared BG, insulin and C-peptide (CP), total cholesterol (TC), triglyceride (TG), high-density lipoprotein cholesterol (HDL-C) and low-density lipoprotein cholesterol (LDL-C) serum levels during a 3 h oral glucose tolerance test (OGTT) in the 3 groups.

**Results:**

Basic overall HbA1c serum concentration percentages were 5.52, 6.33 and 9.76% for the NTG, IGR and T2DM cases. During the OGTT, insulin secretion in the IGR group was almost double that of the T2DM group. CP levels were highest in the IGR patients and OGTT related BG concentrations were highest in the T2DM group followed by IGR, but in the IGR group hyperglycemia was less pronounced than in T2DM patients (*P* <  0.001). Compared to the NGT group, TC, TG and LDL-C serum concentrations were significantly higher (*P* ≤ 0.001) and HDL-C concentrations were significantly lower (*P* ≤ 0.001) in IGR and T2DM cases compared to the NTG group.

**Conclusions:**

IGR led to similar unfavorable blood lipid patterns compared with T2DM patients and an imbalance of insulin and CP serum concentrations during an OGTT.

## Background

Diabetes is a chronic metabolic disease due either to a lack of insulin secretion or a reduced insulin sensitivity, with high blood sugar levels being the main characteristic, and is often associated with fat, protein and electrolyte metabolic disorders and acid-base imbalance. The risk of heart, brain or renal vascular disease is 3 to 5 times higher in diabetes patients than in normal subjects [3, 4, 15]. Previous studies have shown that major vascular complications are already manifest even in subjects with blood glucose levels under the limit for the diagnosis of diabetes [1]. However, a previous study noted that there are considerable ethnic differences regarding insulin sensitivity and β cell function even within Asians [21].

An increase in body mass index (BMI) plays an important role in the progress from IGR to type 2 diabetes mellitus (T2DM) and metabolic disorders may contribute to the development of IGR in non-diabetic subjects [16]. The IGR phase is a transition state between normal glucose tolerance and diabetes, and active intervention can avoid the development of diabetes [23]. A change in diet and increased exercise together with the administration of antihyperglycemic agents such as metformin, acarbose and thiazolidinedione (TZDS), has been shown to prevent or delay the progression from prediabetes to T2DM [3, 4, 15, 25]. However, IGR is often concurrent with high blood lipid or lipid metabolic abnormalities [17] and proposed to be a high pathological status not so different than T2DM [20]. In the present study, we looked for differences in blood glucose and lipid changes in IGR, NGT and T2DM groups to identify lipid metabolism abnormities in a Chinese population.

## Methods

### Subjects

From January 2011 to December 2015, 354 diabetic patients and health check-up subjects visited the Xi’an Ninth Hospital affiliated to Xi’an Jiaotong University and were enrolled in the present study. The patients were divided into 3 groups according to the WHO diagnostic criteria of 1999, including 174 patients (106 men and 68 women) in the T2DM group, aged from 30 to 70 years, who had T2DM for 1 to 20 years, and 112 patients in the impaired sugar regulation (IGR) group, including 52 cases of impaired glucose tolerance (IGT) and 60 cases of impaired fasting glucose regulation (IFG), aged from 30 to 70 years. In addition, 68 healthy subjects aged from 30 to 70 years with normal glucose tolerance (NGT) served as the control group. The criteria for exclusion from the study were: type 1 diabetes; secondary diabetes; primary disease of the heart, brain or kidney; various acute chronic infectious diseases and endocrine disorders; or serious systemic diseases such as malignant tumors. Patients taking statins and fibrates were also excluded.

Diabetes medications included insulin aspart as well as insulin detemir injections (Novo Nordisk Pharmaceutical Co. Ltd.), insulin glargine injections, glimepiride and alogliptin benzoate tablets (Sanofi Pharmaceutical Co. Ltd.), metformin hydrochloride extended release tablets (Merck Pharmaceutical Co. Ltd.) and acarbose tablets (Bayer HealthCare Co. Ltd.). Three days before the OGTT, insulin aspart 30, insulin detemir, insulin glargine, glimepiride and alogliptin were discontinued and medications were switched to an insulin pump containing insulin aspart and metformin or/and acarbose. Insulin administration was also discontinued at least 4 h before the OGTT and no medication was taken before the OGTTs. All OGTTs were conducted only with patients, whose fasting blood glucose was less than 9 mmol/L.

### Diagnostic criteria (1999 WHO)

Diagnostic criteria for T2DM were: diabetes symptoms (polydipsia, polyphagia, polyuria, weight loss, skin itching, blurred vision) plus random blood glucose ≥11.1 mmol/L or fasting glucose ≥7.0 mmol/L or a blood glucose level 2 h after taking glucose ≥11.1 mmol/L. Those subjects without symptoms of diabetes were re-examined on another day. IFG refers to a fasting glucose level ≥ 6.1 mmol/L and ≤ 7.0 mmol/L, and postprandial blood glucose ≤7.8 mmol/L. IGT refers to a fasting glucose level < 6.1 mmol/L and a postprandial blood glucose level ≥ 7.8 mmol/L and ≤ 11.1 mmol/L. All the above blood glucose values were detected in blood plasma. If a patient was recruited already taking glucose-lowering medication and his/her OGTT was completely normal, the diagnosis of T2DM was based on prior use of T2DM medication.

### Medical history and physical examination

Detailed medical histories were taken and physical examinations performed by trained researchers. Data regarding blood pressure, height, weight, waist circumference and hip circumference were recorded in a unified form after which the BMI and waist-to-hip ratio (WHR) were calculated.

### Biochemical index detection

Fasting blood samples were taken from all participants to detect glycosylated hemoglobin (HbA1c), blood glucose (BG), total cholesterol (TC), triglyceride (TG), high-density lipoprotein cholesterol (HDL-C), low density lipoprotein cholesterol (LDL-C)) as well as insulin and C-peptide (CP). All indexes were monitored at 30 min, 60 min, 120 min and 180 min after subjects had orally taken 75 g glucose. The C-peptide is the split product of proinsulin, which is generated when the islet β cells produce an insulin molecule and measurements of insulin and C-peptide can be used to reflect the reserve functional status of islet β cells.

HbA1c (%) was measured in fresh blood samples containing EDTA by using high-performance liquid chromatography (Tosoh Automated Glycohemoglobin Analyzer, Tosoh Corporation, Japan). Plasma glucose, TG, TC, and LDL-C were analyzed enzymatically by using an Auto Biochemical Analyzer (MODULAR-000GS; Roche, Basel, Switzerland). LDL-C was determined by a commercial homogeneous direct measurement method (Reagent: Shanghai Fosun Long March Medical Science Co., Ltd., Shanghai, China). Insulin and C-peptide (CP) were measured with an Automatic Electrochemical Luminescence Analyzer (Cobas 6000, Roche, Basel, Switzerland).

The laboratory variation coefficients of TG, TC, and LDL-C were 4.2, 1.7, and 3.8%, respectively. All blood samples were analyzed within 4 h of collection.

### Insulin resistance and insulin β cell function assessment

A homeostasis model assessment of insulin resistant (HOMA-IR) was calculated as fasting glucose × fasting insulin / 22.5 and the islet β cell function index (homeostasis model assessment of β cell function (HOMA-β)) as 20 × fasting insulin / (fasting blood glucose - 3.5). Insulin sensitivity was calculated using the quantitative insulin sensitivity check index (QUICKI) = 1 / [log fasting insulin (mU/mL) + log fasting glucose (mg/dL) [8]. The Matsuda indices were determined according to previous literature as (10,000/square root of [fasting glucose × fasting insulin] × [mean glucose × mean insulin during OGTT]) [11].

### Statistical analysis

SPSS Statistics for Windows (ver. 17.0, SPSS Inc., Chicago, US) was used for all statistical analyses. Measurement data are displayed as the mean ± standard error. The normal distribution of each variable was tested; comparison of variables in a normal distribution was performed using variance analysis and the Q test. HOMA-IR and HOMA-β data are not normally distributed and its natural logarithm was calculated for comparison with a rank sum test. Correlation between the variables was analyzed by a linear correlation analysis and multiple linear regression analysis. *P* <  0.05 was considered to be a statistically significant result.

## Results

### Comparison of baseline data among the 3 groups

Waist circumference, WHR and BMI as well as TG, TC, LDL-C, insulin and C-peptide were highest in the IGR group. HOMA-IR values were highest and QUICKI values lowest in T2DM patients. The Matsuda index was highest in the NGT, followed by the IGR and lowest in the T2DM group. Basic HbA1c values were 5.52 ± 0.44, 6.33 ± 0.40 and 9.76 ± 2.79 in the NTG, IGR and T2DM patients, respectively (Table 1).

**Table 1:** Basic information about the IGR, NGT and T2DM groups

	NGT (*n* = 68)	IGR (*n* = 112)	T2DM (*n* = 174)	*P*-value NGT vs IGR	*P*-value NGT vs T2DM	*P*-value IGR vs T2DM
Male	42	66	92			
Female	26	46	82			
Age (years)	53 ± 10	53 ± 9	55 ± 10	1.000	0.163	0.087
BMI (kg/m^2^)	23.88 ± 2.59	25.91 ± 3.21	24.52 ± 3.08	< 0.001	0.131	0.001
Waist circumference (cm)	82.09 ± 8.47	92.60 ± 10.53	90.43 ± 7.68	< 0.001	< 0.001	0.045
WHR	0.83 ± 0.07	0.93 ± 0.06	0.91 ± 0.05	< 0.001	< 0.001	0.003
SBP (mmHg)	128.54 ± 15.71	132.94 ± 12.99	135.96 ± 16.37	0.044	0.002	0.101
DBP (mmHg)	76.46 ± 11.55	81.47 ± 8.77	81.43 ± 9.36	0.001	0.001	0.971
TG (mmol/L)	1.38 ± 0.71	1.95 ± 1.02	1.78 ± 0.74	< 0.001	0.001	0.104
TC (mmol/L)	4.99 ± 0.99	5.82 ± 0.86	5.48 ± 1.10	< 0.001	0.002	0.006
HDL-C (mmol/L)	1.35 ± 0.39	1.27 ± 0.24	1.30 ± 0.26	0.090	0.248	0.327
LDL-C (mmol/L)	2.16 ± 0.75	3.21 ± 0.83	3.02 ± 0.82	< 0.001	< 0.001	0.058
FBG (mmol/L)	5.04 ± 0.57	6.08 ± 0.70	8.37 ± 1.56	< 0.001	< 0.001	< 0.001
HbA1c (%)	5.52 ± 0.44	6.33 ± 0.40	9.76 ± 2.79	< 0.001	< 0.001	< 0.001
FINS (mU/mL)	10.50 ± 4.85	11.61 ± 5.90	8.14 ± 5.22	0.193	0.001	< 0.001
C-peptide (μg/L)	3.10 ± 0.86	3.23 ± 1.49	2.12 ± 0.67	0.513	< 0.001	< 0.001
HOMA-IR*	2.42 ± 0.48	2.85 ± 0.53	3.00 ± 0.67	< 0.001	< 0.001	0.046
HOMA-β*	4.78 ± 0.46	4.05 ± 0.33	3.72 ± 0.52	< 0.001	< 0.001	< 0.001
QUICKI *	0.375 ± 0.02	0.356 ± 0.01	0.336 ± 0.01	< 0.001	< 0.001	< 0.001
Matsuda index	14.92 ± 2.86	8.07 ± 1.47	5.87 ± 1.31	< 0.001	< 0.001	< 0.001

*Adjusted for BMI; *BMI* Body Mass Index, *WHR* Waist-to-Hip Ratio, *SBP* Systolic Blood Pressure, *DBP* Diastolic Blood Pressure, *TG* Triglyceride, *TC* Total Cholesterol, *HDL-C* High Density Lipoprotein Cholesterol, *LDL-C* Low Density Lipoprotein-Cholesterol, *FBG* Fasting Blood Glucose, *HbA1c* Hemoglobin A1c, *FINS* Fasting Insulin, *HOMA-IR* Homeostasis Model Assessment-Insulin Resistance index, *HOMA-β* Homeostasis Model Assessment -pancreatic β cell function index, *QUICKI* Quantitative Insulin Sensitivity Check Index

**Fig. 1:**
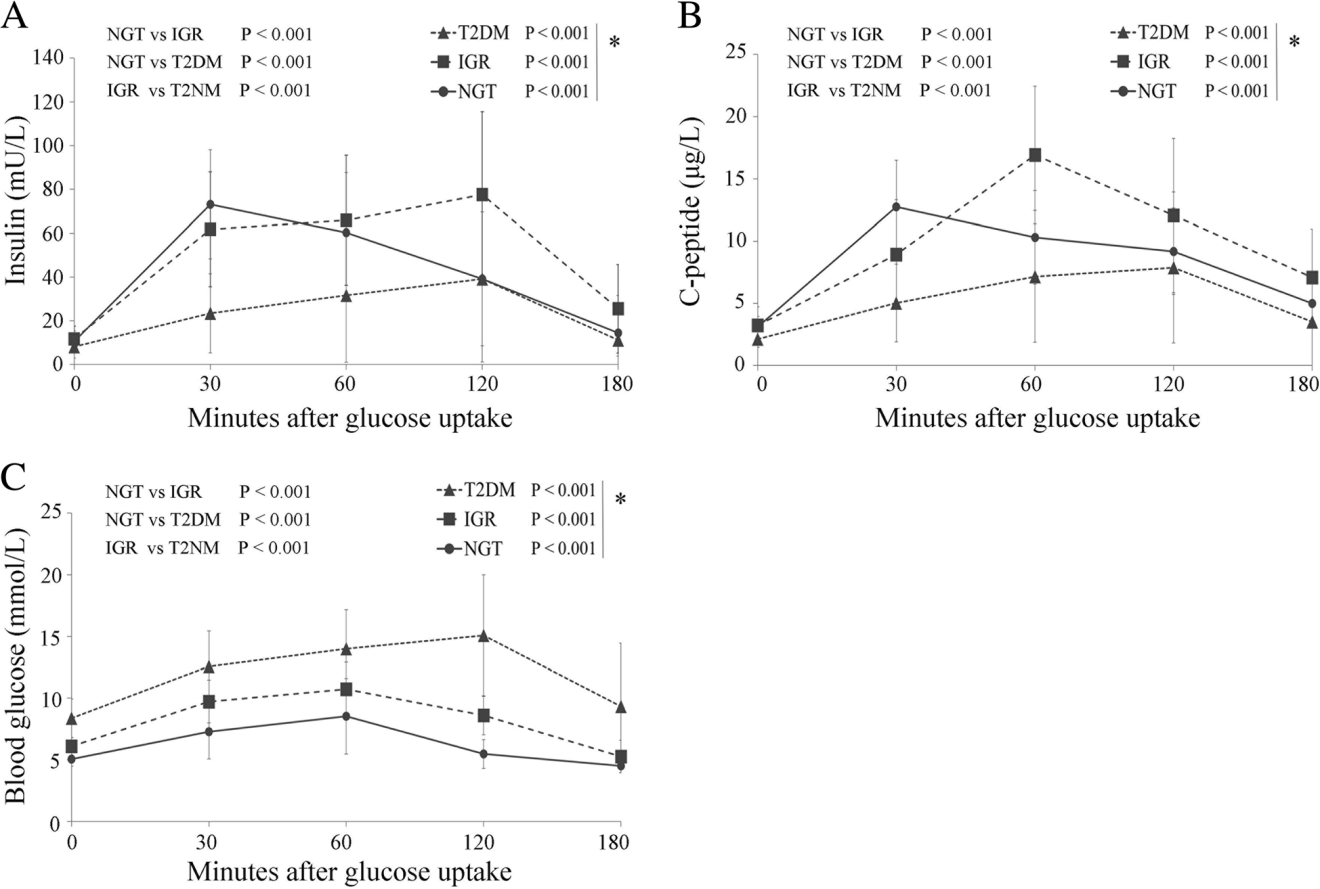
Serum concentrations of **a**) insulin, **b**) CP and **c**) BG in the NGT, IGR and T2DM groups at the indicated time points after the start of the OGTTs. * indicate significance of changes within the time points in the indicated groups

Next, we investigated the levels of insulin and C-peptide during OGTT tests. We found that insulin serum concentrations were significantly different in the 3 groups and reached a peak 30 min after the start of the OGTT in the NGT group, and after 120 min in the T2DM and IGR groups (Fig. 1a), with the areas under the curves (AUC_0_–_180) being_ 7849, 10,417and 4930 for insulin, 1.593, 2015 and 1083 for C-peptide and 1.140, 1539 and 2316 for FBG in the NTG, IGR and T2DM groups.

Similarly, the CP serum concentrations reached their peak levels at 30 min after the start of the OGTT in the NGT group, at 120 min in the T2DM group and after 60 min in the IGR group (Fig. 1b), with the areas under the curves (AUC_0_–_180)_ being 1593, 2015 and 1083 in the NTG, IGR and T2DM groups, respectively.

Thus, insulin metabolism in the IGR group was more similar to that in the T2DM group, although the insulin serum concentration in the IGR group was almost twice that in the T2DM group. The BG level pattern in the IGR group was similar to that in the NGT group, with a decline from 60 min after the start of the OGTT, but the glucose concentrations were significantly higher in the IGR group. The T2DM group had the highest glucose concentrations (*P* <  0.001) with a delay of decline until 120 min after the start of the OGTT (Fig. 1c). The (AUC_0_–_180)_ for BG levels were 1140, 1539 and 2316 in the NTG, IGR and T2DM groups, respectively.

**Fig. 2:**
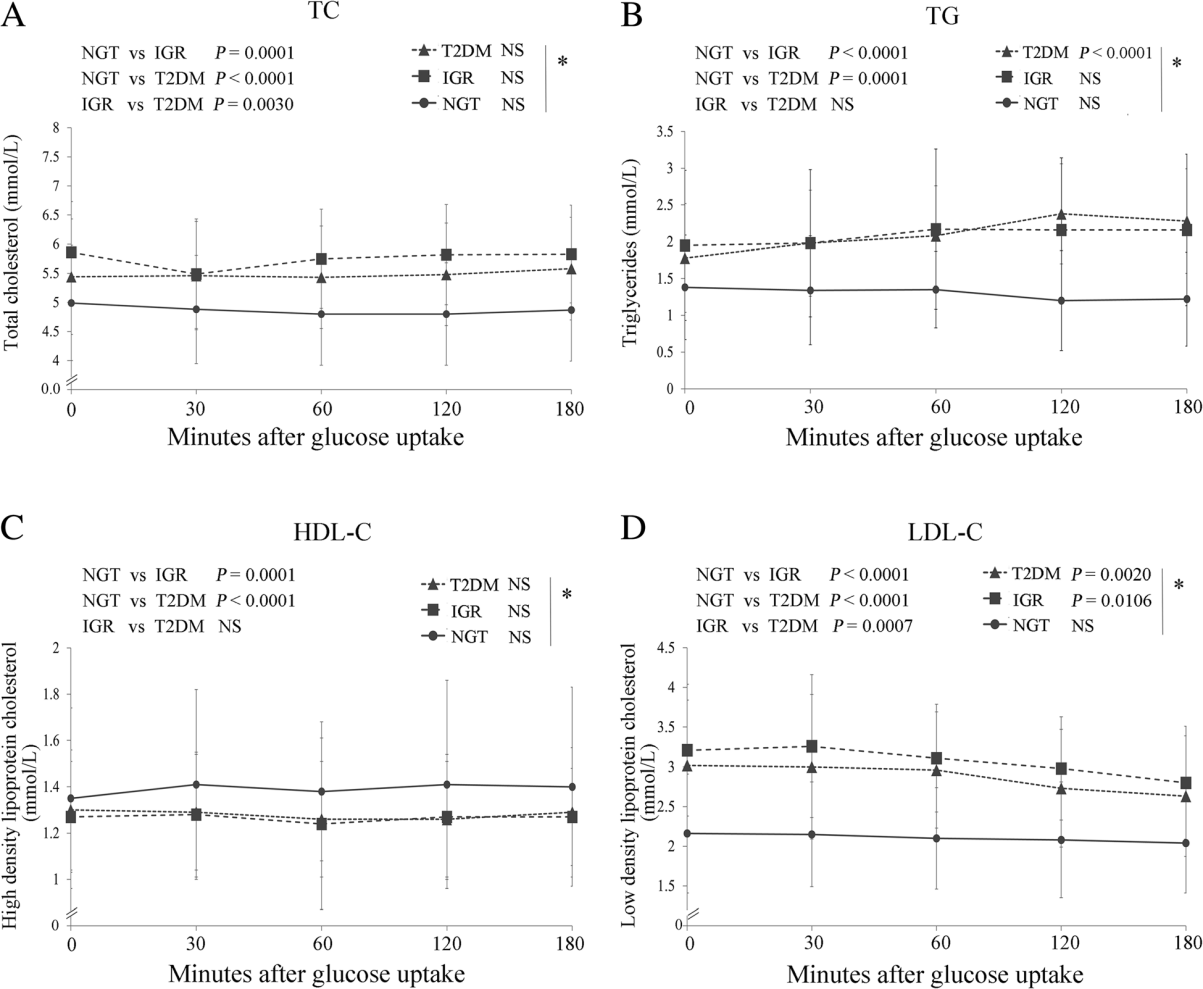
Serum concentrations of **a**) TC, **b**) TG, **c**) HDL-**c** and **d**) LDL-C in the NGT, IGR and T2DM groups at the indicated time points after the start of the OGTTs * indicate significance of changes within the time points in the indicated groups, NS = not significant

TG and HDL-C serum concentrations were similarly enhanced and reduced in the IGR and T2DM compared to the NGT patients (*P* <  0.001), whereas TC and LDL-C levels were significantly highest in the IGR, followed by the T2DM patients (Fig. 2).

## Discussion

IGR refers to the metabolic state of blood glucose levels between a normal and diabetic population, including impaired fasting glucose regulation and impaired glucose tolerance, which can cause tissue or organ structural and functional changes.

According to a previous study, the development of T2DM is a slow process [24]. First, the total number of islet cells increase with normal islet cell structure and gene expression, which can be designated as the islet cell functional compensatory period. Second, a time period is characterized by a slightly raised blood glucose levels, a slow response of the glucose receptor on the surface of the islet cell to glucose stimulation and enhanced insulin secretion. This time period, reflecting impaired glucose regulation and abnormal glucose metabolism, occurs due to the accumulation of insulin and the subsequent higher TG and LDL-C levels, as revealed by the results shown in Figs. 1 and 2. In the present study, we found the highest levels of insulin and CP in the IGR group. The biggest stress reaction of islet β cells in the IGR and T2DM groups occurred 90 min later than in the NGT group, and the highest level of CP release in the IGR patients was 30 min after the NGT and 1 h before the T2DM cases. These findings suggested that there was an imbalance of islet β cell function and CP release in the IGR group and insulin non-responsiveness lead to almost a doubling of the insulin serum concentration, which was also reflected in significantly enhanced BG values compared to the NGT group (Fig. 1c), and may, if not controlled, lead to the serious complications of T2DM [7, 27]. The overall HbA1c serum concentration percentage in the IGR group was 6.33, which is less than the threshold of ≥6.5% for diabetes diagnosis proposed by the American Diabetes Association, but in the range of ≥6% and < 6.5%, which is considered to be a high risk for progression to diabetes [6].

Our study found that waist circumference, WHR and BMI were highest in IGR cases, indicating, in agreement with previous studies, that IGR like T2DM is related to obesity [18]. Blood lipid examinations during the OGTTs revealed that TG, TC and LDL-C levels were all significantly higher in IGR and T2DM patients, whereas HDL-C values were significantly lower compared to the NGT control. These findings are in contrast with a previous study in which only low HDL-C values correlated with hyperglycemia in a Chinese Han population [26].

However, our results indicate that in T2DM and IGR patients compared to non-affected persons a latent dyslipidemia prevails, which is in agreement with previous studies that noted that insulin resistance and dyslipidemia occur in combination or profoundly interact [2, 14, 19, 22]. However, it is not entirely clear whether dyslipidemia leads to insulin resistance or the reverse, since some researchers believe that high triglyceride levels and low plasma HDL-C levels are consequences of insulin resistance [10], whereas others favour the hypothesis that insulin resistance is caused by dyslipidemia [5]. It must be taken into account, that significant differences of blood lipid profiles have been reported for different ethnicities, since low HDL-C values are more frequently found in Asian Indians, followed by central and northern Europeans, Japanese and least in Qingdao Chinese regardless of their glucose categories [28]. Also correlations between insulin sensitivity and insulin response differ between ethnicities with highest insulin sensitivity and lowest insulin response in east Asians compared to Africans and Caucasians [9].

Nevertheless, our data revealed that in the IGR group of the present study, serum lipid concentrations were enhanced and HDL-C concentrations reduced, which has been proposed to be a marker of insulin resistance [12, 13].

## Conclusions

The present study showed that in an OGTT, insulin and C-peptide serum concentrations were higher in the IGR than in the T2DM and NGT groups. In addition, TC, TG and LDL-C were all significantly higher and HDL-C values significantly lower in the IGR and T2DM groups compared to the NGT group.
